# Cellular localization of ROS and NO in olive reproductive tissues during flower development

**DOI:** 10.1186/1471-2229-10-36

**Published:** 2010-02-24

**Authors:** Adoración Zafra, María Isabel Rodríguez-García, Juan de Dios Alché

**Affiliations:** 1 Department of Biochemistry, Cell and Molecular Biology of Plants, Estación Experimental del Zaidín, Consejo Superior de Investigaciones Científicas (CSIC), Profesor Albareda 1, 18008 Granada, Spain

## Abstract

**Background:**

Recent studies have shown that reactive oxygen species (ROS) and nitric oxide (NO) are involved in the signalling processes taking place during the interactions pollen-pistil in several plants. The olive tree (*Olea europaea *L.) is an important crop in Mediterranean countries. It is a dicotyledonous species, with a certain level of self-incompatibility, fertilisation preferentially allogamous, and with an incompatibility system of the gametophytic type not well determined yet. The purpose of the present study was to determine whether relevant ROS and NO are present in the stigmatic surface and other reproductive tissues in the olive over different key developmental stages of the reproductive process. This is a first approach to find out the putative function of these signalling molecules in the regulation of the interaction pollen-stigma.

**Results:**

The presence of ROS and NO was analyzed in the olive floral organs throughout five developmental stages by using histochemical analysis at light microscopy, as well as different fluorochromes, ROS and NO scavengers and a NO donor by confocal laser scanning microscopy. The "green bud" stage and the period including the end of the "recently opened flower" and the "dehiscent anther" stages displayed higher concentrations of the mentioned chemical species. The stigmatic surface (particularly the papillae and the stigma exudate), the anther tissues and the pollen grains and pollen tubes were the tissues accumulating most ROS and NO. The mature pollen grains emitted NO through the apertural regions and the pollen tubes. In contrast, none of these species were detected in the style or the ovary.

**Conclusion:**

The results obtained clearly demonstrate that both ROS and NO are produced in the olive reproductive organs in a stage- and tissue- specific manner. The biological significance of the presence of these products may differ between early flowering stages (defence functions) and stages where there is an intense interaction between pollen and pistil which may determine the presence of a receptive phase in the stigma. The study confirms the enhanced production of NO by pollen grains and tubes during the receptive phase, and the decrease in the presence of ROS when NO is actively produced.

## Background

Both reactive oxygen species (ROS) and nitric oxide (NO) are involved in numerous cell signalling processes in plants, where they regulate aspects of plant cell growth, the hypersensitive response, the closure of stomata, and also have defence functions [1-5]. In *A. thaliana *stigmas, ROS/H_2_O_2 _accumulation is confined to stigmatic papillae and could be involved in signalling networks that promote pollen germination and/or pollen tube growth on the stigma [6]. In addition, the putative presence of ROS in the stigma exudate could be a defence mechanism against microbe attack, similar to the secretion of nectar [6,7]. Several studies have implicated ROS and NO as signalling molecules involved in plant reproductive processes such as pollen tube growth and pollen germination [8-11] and pollen-stigma interactions [6,12]. Low levels of NO was detected by these authors in stigmas, whereas NO was observed at high levels in pollen. An interesting suggestion to explain the biological function of ROS/H_2_O_2 _in stigmas and NO in pollen was proposed by Hiscock and Allen [13], who observed a reduction of these molecules in the stigmatic surface when either pollen grains of NO were artificially added. They propose that the main function of stigmatic ROS/H_2_O_2 _can be defence against pathogens, whereas pollen NO may cause a localized reduction of these molecules, then breaching this defence system. Evidence for the connections between Ca^2+ ^and NO signalling pathways is also beginning to emerge [14-18]. Although there are diverse modes of NO production in plants [4,19], not all of them are regulated by calcium ions.

The presence of numerous specific ROS-related activities (catalases, superoxide dismutases, ascorbate peroxidase, monodehydroascorbate reductase and GSH-dependent dehydroascorbate reductase, peroxidases, glutathione S-transferases) has been characterized in pollen grains [20,21]. Recently, NADPH oxidase activity has been shown to be present at the tip of the pollen tube [10]. However, less is known about these enzymes in the stigma, where only a specific stigma peroxidase has been detected up to date [22]. Most of these studies have been carried out in model species like *Lilium*, *Arabidopsis *and *Petunia*, and in the UK-invading species *Senecio squalidus*. More effort is needed to determine whether the presence of these molecules throughout the reproductive tissues is a general feature of all Angiosperms.

The olive tree (*Olea europaea *L.) has a high economical and social importance in the Mediterranean area. Although several studies are beginning to uncover the details of the reproductive biology in this plant [23,24], much is still unknown. Olive pollination is mainly anemophilous. Paternity tests have revealed a certain degree of self-incompatibility (SI) in several olive cultivars [25,26]. The pistil of the olive tree (*O. europaea *L. c.v. *Picual*) is composed of a two-lobed wet stigma, a solid style and a two-loculus ovary with four ovules. The exudate of the olive stigmatic receptive surface is heterogeneous, including carbohydrates, lipids and proteins in its composition [23,24]. All these structural and cytochemical features of the pistil in olive are in good agreement with the presence of a SI mechanism of the gametophytic type in this plant, in accordance with general consensus and previous observations carried out in olive and other *Oleaceae *species [23,24,27-29].

The purpose of this study was to first approach the possible implications of ROS and NO during flower development and the pollen-pistil interactions in the olive. For this purpose, several of these molecules have been precisely localized in the stigma and the pollen during the main developmental stages of flowering.

## Results

### Developmental stages of olive flowering

**Figure 1:**
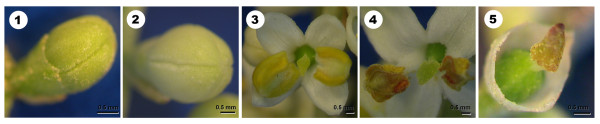
**Developmental stages of the olive flower**. Stage 1: "green bud". Stage 2: "white bud". Stage 3: "recently opened flower". Stage 4: "dehiscent anther". Stage 5: "abscised anthers and petals".

Five major developmental stages were established to better scrutinize flower development in the olive (Figure 1). Very early stages were omitted, as olive flower buds were completely covered by solid trichomes which made dissection very difficult without compromising the integrity of anthers and gynoecium, and therefore altering the presence of ROS/NO. Flower buds at the "green bud" stage (stage 1) had an average size of 2.5 ± 0.2 mm length × 1.7 ± 0.1 mm width. All flower organs were green coloured. This stage lasted for 8 days on the average. At the "white bud" stage (stage 2), the floral buds were 3.3 ± 0.1 mm length × 2.7 ± 0.7 mm width on the average. Petals have changed from green to whitish colour although they were still wrapping the remaining organs into the unopened flower. This stage lasted an average of 4 days. At the "recently opened flower" stage (stage 3), of two days of duration, the four white petals turned out to be separated, leaving the remaining floral structures visible: the anthers coloured in yellow, and the stigma, style and ovary which remained in green colour. At the "dehiscent anther stage" (stage 4), two days long, one or the two anthers became dehiscent, releasing the pollen grains, which also covered the stigma. In the last developmental step (stage 5), anthers and petals were abscised. The apex of the stigma appeared clearly brown-coloured. Only the two first days of this stage were considered.

### Light Microscopy detection of H_2_O_2_

**Figure 2:**
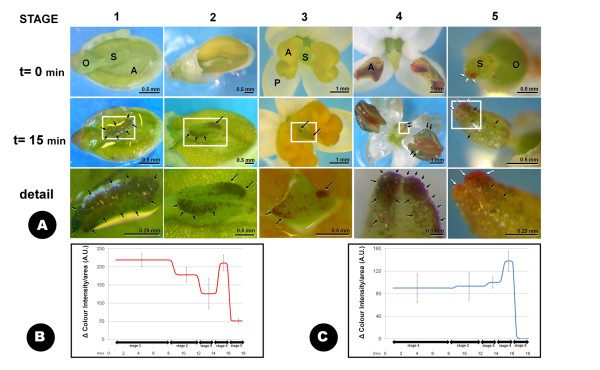
**LM detection of H_2_O_2 _with TMB at different developmental stages of the olive flower**. **A: **the presence of H_2_O_2 _is shown by a dark purple precipitate appearing shortly (c 15 minutes) after the incubation with the appropriate medium (black arrows). This precipitate is clearly distinguishable from the dark brown colour appearing at the latest stages of flower development (white arrows). The last row of pictures shows some details of the labelling at larger magnification. **B: **quantification of the labelling intensity detected over the stigma surface. **C: **quantification of the labelling intensity over the anther surface. Both the average and the standard deviation displayed in the graphs correspond to the measurement of a minimum of nine images, on three independent experiments. A: anther; AU: arbitrary units; O: ovary; P: petal; S: stigma.

Ligh microscopy (LM) detection of H_2_O_2 _with TMB (3,5,3',5'-tetramethylbenzidine-HCl) solution was assayed in olive flowers during different stages of its development (Figure 2). Once the chemical was added, a progressive change of colour was observed in both the stigmas and the anthers, as the result of the presence of a dark purple precipitate. Neither the style nor the ovary tissues were coloured. The appearance and localization of H_2_O_2 _was not homogenous in all the developmental stages studied: during stage 1, the precipitate started to accumulate at the very distal part of the stigma shortly after de beginning of the treatment, spreading throughout the borders of the stigma until covering almost all its surface. Anthers showed no change of colour at the green bud stage. White buds stigmas (stage 2) also started to be coloured in the distal part of the stigma. However, the progressive appearance of the precipitate was relatively slower and finally covered less area of the stigma and showed lower intensity than in stage 1, becoming limited to the peripheral regions of the stigma. As in stage 1, no H_2_O_2 _was detected in the anthers in this stage. The stigmas of the newly opened flowers (stage 3) started to be coloured soon after the initiation of the histochemical staining. In this case, the presence of the purple precipitate was restricted to the distal part of the stigma and to some small spots on the remaining stigma surface.

At stage 4, the distribution of the coloured precipitated over the stigma was even more limited, focusing into the stigma two-lobed apex only. At this stage we detected an intense purple coloration corresponding to the massive presence of H_2_O_2 _in the dehiscent anthers even after 5 minutes of treatment. Finally, over the last stage (stage 5), very little purple colour appeared in the stigma, even after long periods of incubation with the reagent. As described above, anthers are absent at this stage.

### Confocal Laser Scanning Microscopy detection of ROS

**Figure 3:**
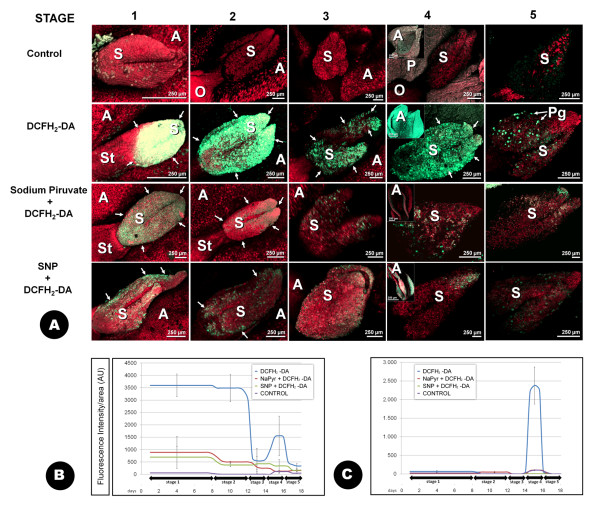
**Low-magnification CLSM detection of H_2_O_2 _with DCFH_2_-DA at different developmental stages of the olive flower**. Projections of section stacks **A: **the presence of H_2_O_2 _is shown by green fluorescence (arrows), which is clearly distinguishable from the tissues autofluorescence, showed here in red colour. Co-localization of both fluorescence sources results in yellow colour. Three different treatments are displayed (DCFH_2_-DA alone or in combination with sodium pyruvate or SNP), as well as untreated samples (control). **B: **quantification of the fluorescence intensity owing to DCFH_2_-DA under the different treatments over the stigma surface. **C: **the same over the anther surface. Both the average and the standard deviation displayed in the graphs correspond to the measurement of a minimum of nine images, on three independent experiments. A: anther; AU: arbitrary units; O: ovary; P: petal; Pg: pollen grain; S: stigma; St: style.

**Figure 4:**
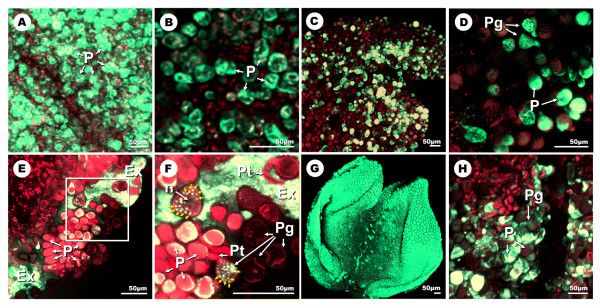
**High-magnification CLSM detection of H_2_O_2 _with DCFH_2_-DA at different developmental stages of the olive flower**. **A and B: **projections of section stacks of the stigma surface at stages 1 and 3, respectively. The fluorescence localizes in association with the stigmatic papillae. **C and D: **optical section -and an enlarged view- of the stigmatic surface in an area lacking exudates at stage 4. **E and F: **optical section -and an enlarged view- of the stigmatic surface at stage 4. Green fluorescence extensively localizes in the exudate, as well as in stigmatic papillae and in small organelles inside some pollen grains (yellow arrows). **G: **projection of section stacks of the anther surface at stage 4. **H: **projection of section stacks of the stigma surface at stage 5. Fluorescence remains associated to the papillae and the pollen grains. Ex: exudate; n: nuclei; P: papillae; Pg: pollen grain; Pt: pollen tube.

The DCFH_2_-DA (2',7'-dichlorodihydrofluorescein diacetate) fluorochrome was used to detect ROS by Confocal Laser Scanning Microscopy (CLSM). Low magnification CLSM allowed the observation of both stigmas and anthers at stages 1, 2 and 3 whereas they were observed separately at stage 4 (Figure 3A). The presence of these chemicals produced a green fluorescence in the stigma and the anthers, which showed different degrees of intensity depending on the stages analyzed (Figure 3B, C). Although the fluorescence was present all through the stigma surface, it was slightly more intense at the distal side of the stigma (the apex of both stigma lobules) than in the basal region of the stigma. The tissue situated between both stigma lobules frequently appeared unlabelled. No fluorescence over the background or the control experiments was detected in the tissues of the ovary or the style at any of the stages analyzed. Autofluorescence of the floral tissues was recorded in red. Stigmas at the stage 1 exhibited the greatest relative intensity of fluorescence per area analysed, in comparison with other developmental stages (Figure 3A,B; additional file 1). High magnification CLSM images of the stigma at the same stage showed the fluorescence to localize in association with the stigmatic papillae present throughout the stigma surface. (Figure 4A).

At stages 2 and 3, stigma size was considerably larger than at the previous stage. Although the distribution of fluorescence was similar to the previous stage, a dramatic decrease in the fluorescence intensity detected on the stigmatic surface was measured (Figure 3B; additional files 2 and 3). Similarly to stage 1, fluorescence concentrated in the stigmatic papillae at these stages (Figure 4B). The stage 4 was characterized by the presence of the stigmatic exudate, which was particularly visible when high magnification observations were carried out. This stigmatic exudate resulted to be intensely fluorescent (Figures 4C and 4D). Pollen grains over the surface of the stigma were observed from stage 3 onwards, and were easily identified even at low magnification (Figure 3A), due to their high levels of fluorescence. At high magnification, fluorescence was in some cases located in small individualised organelles clearly visible inside the pollen grains when observed in single optical sections by CLSM (additional file 4). At this stage, the dehiscent anthers which until now had remained practically free of fluorescence became intensely stained (Figures 3A,B, 4E; additional file 5). Finally, the fluorescence became restricted to the pollen grains over the surface of the stigma at stage 5 (Figure 4F).

The incubation of the samples with the H_2_O_2 _scavenger Na-pyruvate, prior to the treatment with the fluorochrome [6], resulted in a substantially lower intensity of the fluorescence in all the stages and the floral organs assayed (Figure 3A). A similar reduction in the overall levels of fluorescence intensity was observed when the samples were treated with SNP (sodium nitroprusside), a NO donor (Figure 3A). In both cases, the intensities of the residual fluorescence were practically identical to those of the untreated -control- samples (Figures 3A and 3B).

### CLSM detection of O_2_^.-^

**Figure 5:**
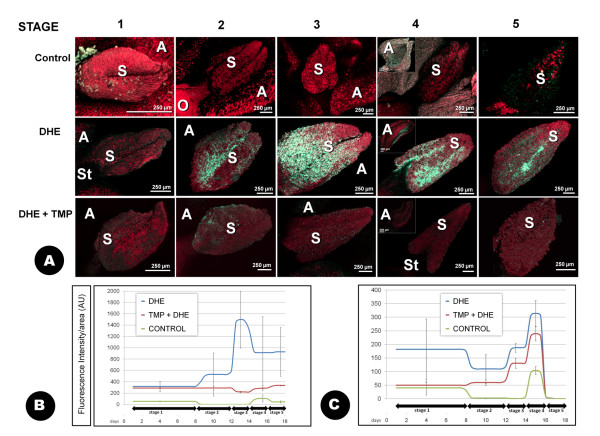
**Low-magnification CLSM detection of superoxide anion (O_2_^.-^) with DHE at different developmental stages of the olive flower**. Projections of section stacks **A: **the presence of O_2 _^.- ^is shown by green fluorescence, which is clearly distinguishable from the tissues autofluorescence (red colour). Two different treatments are displayed (DHE alone or in combination with TMP), as well as untreated samples (control). **B: **quantification of the fluorescence intensity owing to DHE under the different treatments over the stigma surface. **C: **the same over the anther surface. Both the average and the standard deviation displayed in the graphs correspond to the measurement of a minimum of nine images, on three independent experiments. A: anther; AU: arbitrary units; O: ovary; S: stigma; St: style.

**Figure 6:**
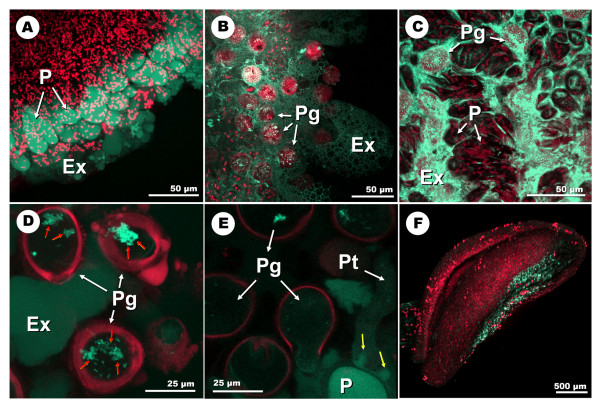
**High-magnification CLSM detection of superoxide anion (O_2_^.-^) with DHE at different developmental stages of the olive flower**, **A: **projection of section stacks of the stigma surface at stage 3. The fluorescence localizes in association with the stigmatic papillae. **B: **stacks projection of the surface of the distal area of the stigma at stage 4. **C: **stacks projection of the surface of the central area of the stigma at stage 4. Note the differences in both the texture of the exudate, and the intensity of the labelling. **D: **optical section of several pollen grains on the stigmatic surface at stage 4. Several clusters of pollen organelles are intensely labelled (red arrows). **E: **optical section of several pollen grains germinating on the stigmatic surface at stage 4. The cytoplasm of the pollen tube appears weakly labelled. However the fluorescence becomes more intense in the contact areas between the pollen tube and the papillae (yellow arrows). **E: **projection of section stacks of the anther at stage 4. Fluorescence localizes in the stomium. The pollen grains show red autofluorescence. Ex: exudate; p: papillae; Pg: pollen grain; Pt: pollen tube.

The incubation of the samples with the DHE (dihydroethidium) fluorophore produced green fluorescence in the presence of O_2 _^.- ^when compared to the control samples (Figures 5A, B). Autofluorescence of both the anthers and the gynoecium was recorded in red. The fluorescence was located in the stigma, mainly at stages 2 to 5, with a maximum of intensity at stage 3 (Figure 5A, B; additional files 6, 7). In this case, the fluorescence was centred at the basal and central region of the stigma, with the apex of both stigma lobules practically unlabelled. The equivalent samples previously incubated with the O_2 _^.- ^scavenger TMP (4-hydroxy-2,2,6,6-tetramethylpiperidine-1-oxy) [30] displayed much reduced fluorescence intensity all over the stigma (Figure 5A). No relevant fluorescence was detected in either the ovary or the style. The anthers presented high levels of fluorescence, particularly at stage 4 (Figure 5C). Images at higher magnification allowed us to determine that fluorescence was particularly evident in particular areas of the anther corresponding to the stomium (Figure 6F; additional file 8). The observation of the samples at high magnification also allowed us to allocate the signal in the stigma mainly to the stigmatic papillae (Figures 6A, E), the exudate and the pollen grains and pollen tubes (additional file 9). Conspicuous differences in the exudate texture and fluorescence intensity were detected between the distal area of the stigma (Figure 6B), and the basal/central area (figure 6C). The pollen grains attached to the stigma exhibited intensely labelled particles or organelles frequently grouped in clusters in the pollen cytoplasm (Figure 6D). Pollen tubes on the surface of the stigma also showed a weak labelling in their cytoplasm, which increased in intensity in the area of the pollen tube in close contact with the stigmatic papillae and the exudates (Figure 6E).

### CLSM detection of NO

**Figure 7:**
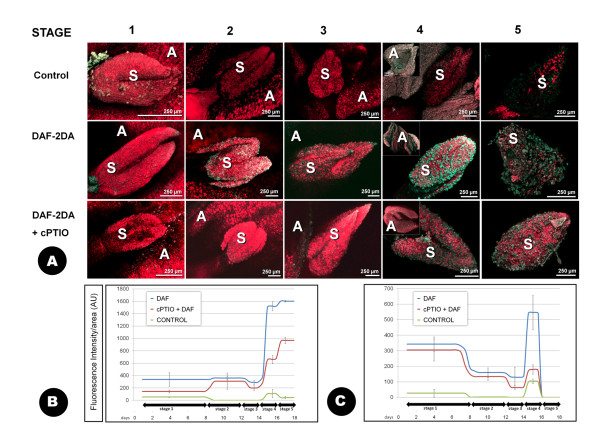
**Low-magnification CLSM detection of NO with DAF-2 DA at different developmental stages of the olive flower**. Projections of section stacks **A: **the presence of NO is shown by green fluorescence (arrows), which is clearly distinguishable from the tissues autofluorescence, showed here in red colour. Co-localization of both fluorescence sources results in yellow colour. Two different treatments are displayed (DAF-2 DA alone or in combination with cPTIO), as well as untreated samples (control). **B: **quantification of the fluorescence intensity owing to DAF-2 DA over the stigma surface. **C: **the same over the anther surface. Both the average and the standard deviation displayed in the graphs correspond to the measurement of a minimum of nine images, on three independent experiments. A: anther; AU: arbitrary units; O: ovary; P: petal; Pg: pollen grain; S: stigma; St: style.

**Figure 8:**
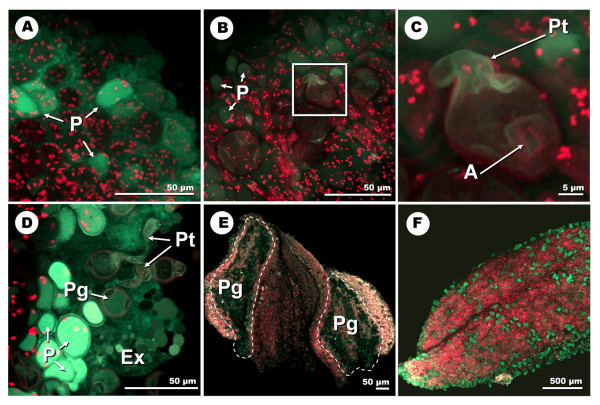
**High-magnification CLSM detection of NO with DAF-2 DA at different developmental stages of the olive flower**. **A: **projection of section stacks of the stigma surface at stage 3. The fluorescence localizes in association with the stigmatic papillae. **B and C: **projection of section stacks -and an enlarged view- of the stigmatic surface at the end of stage 3. Green fluorescence labels the stigmatic papillae and the pollen surface, mainly the apertural region and the emerging pollen tube. **D and E: **optical section -and an enlarged view- of the stigmatic surface at stage 4. NO extensively accumulates in the stigmatic papillae, and in the pollen grains, the pollen tubes and the exudate. **F: **projection of section stacks of the dehiscent anther surface at stage 4. NO labelling occurs in the dehiscent loculi, associated to the numerous pollen grains. Ap: aperture; Ex: exudate; p: papillae; Pg: pollen grain; Pt: pollen tube.

The presence of NO in the olive floral organs was examined by using the DAF-2 DA (2',7'-dichlorodihydrofluorescein diacetate) fluorochrome by CLSM. As it also happened with the DCFH_2_-DA and DHE fluorophores, fluorescence was not observed to occur over the background or the control experiments in the tissues of the ovary or the style at any of the stages analyzed (Figure 7A). Autofluorescence in these tissues was documented in red. Fluorescence was practically negligible over the developmental stages 1, 2 and most of the stage 3, to rise at stage 4, coincidentally with the presence of numerous pollen grains over the stigma surface (Figure 7A, B). At this "dehiscent anther" stage, fluorescence accumulated for the most part at both tips of the two-lobed stigma. The samples treated with cPTIO (2-(4-carboxyphenyl)-4,4,5,5-tetramethylimidazoline-1-oxyl-3-oxide) prior to the incubation with NO showed comparatively reduced levels of fluorescence in all stages studied (Figure 7A). Detailed localization at higher magnification showed that NO started in fact to accumulate at the very end of stage 3, partially in the stigmatic papillae, and mainly in both the apertural regions and the pollen tubes of the scarce pollen grains landed on the stigma surface at this stage (Figure 8A-C; additional files 10, 11). It is at stage 4 when NO was extensively localized in the stigmatic papillae, the pollen tubes and apertures of the numerous pollen grains settled on the stigma. The stigmatic exudate, when present, was also intensely fluorescent. (Figure 8D; additional files 12, 13). The anthers only displayed relevant labelling at stage 4 (Figure 7C), in the form of high levels of autofluorescence and signal co-localization at the stomium. The pollen grains inside the sacs were also fluorescent (Figure 8E; additional file 14). Finally, at stage 5, only residual fluorescence was detected in association with the remaining pollen grains (Figure 8F).

## Discussion

**Figure 9:**
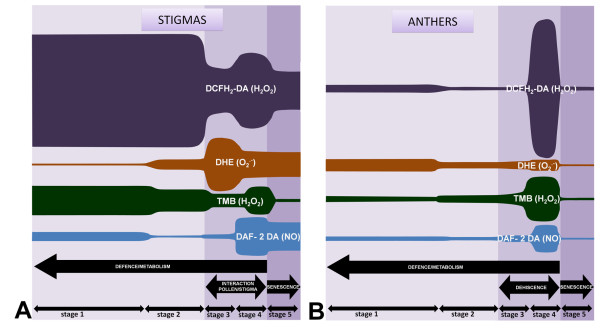
**Summary diagram of the overall presence of ROS and NO in the olive stigma and anther**. **A: **diagram showing the relative abundance of ROS and NO in the stigma at the different developmental stages, as the result of the different histochemical determinations, and proposed functions of these species in the stigma physiology. **B: **the same in the anther.

The present study confirms that the olive tree shares several features with other Angiosperms, as regard to the presence of ROS and NO in reproductive tissues. The first of these features is that H_2_O_2 _is the most prominent ROS in the olive stigma, at least in early stages (1-3). This conclusion is the result of the application of the same criteria already described by [6], mainly the reduction in DCFH_2_-DA fluorescence after the application of the scavenger sodium pyruvate, the strong reaction of the stigmas to TMB (with a practically identical distribution of the labelling by TMB and DCFH_2_-DA), and the relative low presence of other ROS and NO in these stages (as showed by the DHE and DAF-2 DA fluorophores) (Figure 9). The average level of DCFH_2_-DA fluorescence in olive stigmas slightly decreases at stages 3-4, where pollen grains adhere and emit pollen tubes over the stigma. DCFH_2_-DA fluorescence is also notoriously reduced after the addition of SNP, a NO donor. This observation is similar to those described for *Senecio squalidus *[6]. Although olive pollen and pollen tubes are clearly demonstrated in this paper to be major sources of NO, our results do not provide a causal link between NO generated by pollen and this decrease in H_2_O_2 _levels. This and some other possibilities of signalling cross-talk between pollen and stigma have yet to be investigated. This NO production by pollen has now being reported in a number of plant species [8-11,31], and has been connected with the regulation of the rate and orientation of pollen tube growth at the pollen tube tip. Moreover, a possible link between production of NO and nitrite to pollen-induced allergic responses has been proposed [31]. In the case of olive pollen, (a highly allergenic source in Mediterranean countries), further investigation regarding the putative interaction between pollen-produced NO and the immune system is also needed.

The present study is the first to report the presence and distribution of ROS and NO in plant reproductive tissues in a developmental manner. The differential presence of ROS/NO throughout stages 1-5 is likely to correspond to different physiological scenarios. The massive presence of ROS/H_2_O_2 _in the stigma at early stages of flower development (stages 1 and 2) will doubtfully reflect the presence of a receptive phase in the stigma, as flowers at these stages are still unopened, and temporally far from pollen interaction. In this context, some other hypotheses should be taken into account: high levels of ROS/H_2_O_2 _may be generated as the result of the high metabolic activity of the stigmatic papillae and the surrounding tissues, which start to accumulate starch and lipid materials as well as pectins, arabino-galactan proteins and many other components integrating not only the stigma tissues, but also the stigma exudate and a clearly distinguishable cuticle [23,24]. Major differences in starch content have been recently described between staminate and hermaphrodite flowers in the olive tree. Differences in pistil development between these two types of flowers have been related to differences in their sink strength [32]. ROS are likely required for cell expansion during the morphogenesis of the stigma, as has been widely reported for other organs such as roots and leaves [33]. H_2_O_2 _is likely to participate in the peroxidation reactions driven to the formation of the cells walls and many other metabolic reactions, and its levels are tightly regulated by peroxidases, some of them stigma-specific [12,22]. On the other hand, ROS/H_2_O_2 _may also have a putative role in flower defence functions at these early stages. Olive flowers are tightly closed at the very early stages of flower development and until stages 1-2. Many of flower organs are protected by numerous trichomes (Rejón et al., unpublished results), which physically protect them from both desiccation and biotic stresses. High levels of ROS may represent an additional barrier to several pathogens which may include bacteria, fungi and even insects, in a similar manner than in nectar (as widely reviewed by [6,12]).

Once we progress into flower development, different types of interactions start to occur: when the receptive phase of the stigma is reached, high levels of ROS/H_2_O_2 _may harm the pollen grains/pollen tubes growing at the stigma surface. Numerous studies have reported to date the presence of enhanced levels of peroxidase activity in Angiosperm stigmas at maturity [34-37]. Providing that olive stigmas behave similarly, a putative increase in peroxidase activity is therefore likely to take place in olive stigmas at stages 3-4. Peroxidases reduce H_2_O_2 _to water while oxidizing a variety of substrates including glutathione, ascorbate and others. Therefore, they are important enzymatic components of the ROS-scavenging pathways of plants [33]. These high levels of peroxidase activity would be responsible for the observed decrease in the levels of ROS/H_2_O_2_occurred at the later stage, coincidentally with the enhanced receptivity of the stigma to pollen. A forthcoming step in this research is therefore to determine whether this described reduction in the levels of ROS/H_2_O_2 _at the receptive phase is a general feature of Angiosperm stigmas.

Much is still to learn about the source of the described ROS/H_2_O_2 _and NO in the plant reproductive tissues, as showed in this paper. In pollen, plasma membrane-localized NADPH oxidase (NOX) has been described as an active source of superoxide, needed to sustain the normal rate of pollen tube growth in *Nicotiana *[10]. This O_2 _^.- ^readily forms other ROS including H_2_O_2 _and HO^. ^either spontaneously or by the intermediation of other enzymes involved in oxygen metabolism. In the olive pollen, different isoforms of superoxide dismutase (SOD), with extracellular and cytosolic localization have been described [38], and there is clear evidence of the presence of NOX activity (Jiménez-Quesada et al., unpublished observations). However data regarding the stigma tissues are still lacking. In the olive leaves, the presence of different SOD forms has been described [39]. In these tissues, recycling of NADPH by different enzymes, including glucose-6-phosphate dehydrogenase, isocitrate dehydrogenase, malic enzyme and ferredoxin-NADP reductase seems to have an important role in controlling oxidative stress caused by high-salt conditions in olive somatic tissues [40]. As regards to NO production, both NO synthase (NOS) and nitrate reductase activities are considered putative enzymatic sources for NO in pollen, although the presence of other enzymatic sources cannot be excluded [41]. Even though the presence of L-arginine- dependant NOS activity in plant tissues is widely accepted, the identification of the enzyme responsible for this nitric oxide generation is still a matter of controversy [42]. Therefore, much effort is still necessary to characterize these systems in the reproductive tissues of the olive and other Angiosperms. In addition, many of the ROS and NO can be generated in multiple cellular localizations. Peroxisomes have been described as subcellular organelles particularly active in the generation of these signal molecules [43,44]. Further research in order to characterize these organelles in the olive reproductive tissues should be carried out. The extreme ability of these molecules to diffuse may lead to the localization of ROS and NO in some areas as described here, for example, the stigmatic exudate.

The superoxide anion (O_2 _^.-^) is the only detected ROS having a slight increase over the stages 3/4 in the stigma (Figure 9). The rise in the levels of this species can be attributed to the massive presence of pollen grains and growing pollen tubes over the surface of the stigma at these stages, with putatively high rates of NOX activity [10]. In addition, a reduction in the activity of SOD forms can also occur.

The occurrence of ROS/NO at stage 5 of the stigma is coincident with the presence of morphological features indicating senescence of this structure. Decay in plant antioxidant capacity has been described at the terminal phase of senescence for different plant organs, which is frequently coincident with increased release of ROS [45,46]. In *Arabidopsis *flowers, senescence has been connected with low levels of ascorbic acid and therefore alterations of the endogenous levels of both giberelic and abscisic acid [47]. In addition to hormonal imbalance, numerous modifications in the expression of senescence associated genes (SAGs) have been described [48]. Many of these gene products include antioxidant barriers, and thus an increase of the ROS present in the senescent floral organs is likely to occur. Whether this can be considered a mechanism for apoptosis or programmed cell death (PCD) is still a matter of controversy [47-49].

ROS/NO maintain steady low levels in the anther tissues until stage 4, in which a rapid increase takes place (Figure 9B). At this stage, release of mature pollen is produced by breakdown of the anther cells at the stomium, a specialized structure situated at the side of the anthers. Dehiscence of the anther involves a number of PCD mechanisms involving degeneration of the endothecium and the surrounding connective tissues, and selective cytotoxin ablation of the stomium [50]. These changes lead to massive ROS release at this stage, whereas NO is mainly produced by the mature pollen grains.

## Conclusion

Conspicuous changes in the distribution and the proportion of different ROS/NO occur in the reproductive tissues of the olive throughout flower development. These changes correspond to different physiological circumstances (defence, metabolism, signalling...) and reveal the complex interrelationships taking place between the plethora of enzymatic activities involved in their production, the high number of potential substrates and products involved in their metabolism, and the presence of complex signalling pathways. Most changes in ROS occur at stages 3-4, coincidentally with the presence of high levels of NO. Therefore, special attention has to be addressed in the future to the different ROS/NO-signalling pathways present in plant reproductive tissues [51].

## Methods

### Plant material

*Olea europaea *flowers (cv. Picual) at different stages were obtained from adult olive trees growing at the Estación Experimental del Zaidín (Granada, Spain) over the blooming period (fifteen-twenty days throughout the months of May-June). Five different stages were differentiated attending to macroscopic differences. Flowers at the developmental stages 3 to 5 were directly used for ROS and NO determinations. However, flower buds (stages 1 and 2) were dissected by gently removing one of the anthers and the associated petals in order to gain visual access and to allow the contact of chemicals with the gynoecium and the remaining anther.

### Light microscopy

H_2_O_2 _was detected by using the H_2_O_2 _indicator dye TMB (Sigma). Dissected buds or complete flowers at the different stages were soaked in a solution containing 0.42 mM TMB in Tris-acetate, pH 5.0 buffer [52]. The appearance of blue colour was monitored at different times after the initiation of the incubation in a multi-purpose zoom microscope Multizoom AZ-100 (Nikon Instruments Company). Images were gathered with a Nikon Coolpix 4500 digital camera with a resolution of 2272 × 1704 dpi after 15 minutes of incubation (no substantial changes were further observed after that time).

### Confocal Laser Scanning Microscopy

ROS were detected using the fluorescent indicator dye DCFH_2_-DA (Calbiochem). Dissected floral buds or complete flowers were immersed in 50 μM DCFH_2_-DA in MES (2- [N-morpholino]ethanesulfonic acid)-KCl buffer (5 μM KCl, 50 μM CaCl_2_, 10 mM MES, pH 6.15) for 10 minutes followed by a wash step in fresh buffer for 15 minutes and then observed at the confocal microscope. Parallel sets of floral buds/complete flowers at equivalent stages were treated with a) 1 M sodium pyruvate (Sigma-Aldrich) in MES-KCl buffer for 30 min, or b) 500 μM SNP (Sigma-Aldrich) in MES-KCl buffer prior to the treatment whit DCFH_2_-DA as above. Negative controls were treated with MES-KCl buffer only [6].

The presence of the superoxide anion (O_2 _^.-^) was analysed as above by incubating the samples 30 minutes in a 20 μM solution of the fluorophore DHE (Sigma) in Tris-HCl buffer (10 mM, pH 7.4). Equivalent samples were treated with the O_2 _^.- ^scavenger TMP (Calbiochem) in Tris-HCl buffer (10 mM, pH 7.4) for 60 minutes, prior to the treatment with DHE (modified from [30]).

The NO indicator dye DAF-2 DA (Calbiochem) was used to detect NO in flowers. Dissected buds or complete flowers were immersed in MES/KCl pH 6.15 for 10 min, transferred to 10 μM DAF-2 DA for 10 min, followed by a wash step (with MES/KCl buffer) for 15 min and then observed in the microscope [6]. Parallel sets of samples were treated the same, although they were previously incubated for 1 hour with the NO-scavenger cPTIO (Sigma) in a concentration of 400 μM in Tris-HCl 10 mM, pH 7.4 [30]. Negative controls were treated with MES-KCl buffer only instead of DAF-2 DA.

Observations were carried out in a Nikon C1 confocal microscope using an Ar-488 laser source and different levels of magnification (20× to 60×). Small pinhole sizes (30 μm) were used even in combination with low-magnification, dry-objectives. Multiple optical sections were captured and processed to generate 3-D reconstructions of the whole stigma surface. 3-D reconstructions of small areas of the stigma surface were also generated from high-magnification immersion-objectives. The fluorescent signal was obtained exclusively in the range of the 515-560 nm emission wavelengths with both fluorochromes, and was recorded in green colour. Autofluorescence (mainly due to the presence of chlorophyll and other pigments and secondary metabolites) was isolated and displayed in red. For each fluorochrome, identical settings were used for image capture in both control/test experiments in order to ensure reproducibility and accurate quantification.

### Colour and fluorescence quantification

The intensity of both the dark purple-coloured precipitate and the green fluorescence was quantified for each organ at the different stages studied by using the Nikon EZ-C1 viewer (3.30) software. Both average and standard deviation were calculated after measurement of a minimum of nine images corresponding to three independent experiments.

For quantification of the dark purple-coloured precipitate, an independent subtraction of the background was performed for each sample. For this purpose, images of the samples were also captured prior to the addition of the chemicals.

## Abbreviations

AU: arbitrary units; CLSM: confocal laser scanning microscopy; cPTIO: 2-(4-carboxyphenyl)-4,4,5,5-tetramethylimidazoline-1-oxyl-3-oxide; DAF-2 DA: diaminofluorescein diacetate; DCFH_2_-DA: 2',7'-dichlorodihydrofluorescein diacetate; DHE: dihydroethidium; LM: light microscopy; MES: 2-(N-morpholino)ethanesulfonic acid; NOX: nicotinamide adenine dinucleotide phosphate-oxidase; PCD: programmed cell death; ROS: reactive oxygen species; SAG: senescence associated gene; SI: self-incompatibility; SNP: sodium nitroprusside; SOD: superoxide dismutase; TMB: 3,5,3',5'-tetramethylbenzidine-HCl; TMP: 4-hydroxy-2,2,6,6-tetramethylpiperidine-1-oxy.

## Authors' contributions

JDA and MIR conceived the study. JDA and AZ designed and carried out the experiments. AZ performed quantification. The three authors discussed the results and prepared the manuscript. All authors read and approved the final manuscript.

## Supplementary Material

Additional file 1 Animated 3-D reconstruction of CLSM detection of ROS in a flower at stage 1 with DCFH_2_-DA at low magnification. Click here for file

Additional file 2 Animated 3-D reconstruction of CLSM detection of ROS in a flower at stage 2 with DCFH_2_-DA at low magnification. Click here for file

Additional file 3 Animated 3-D reconstruction of CLSM detection of ROS in a flower at stage 3 with DCFH_2_-DA at low magnification. Click here for file

Additional file 4 z-Animated 3-D reconstruction of CLSM detection of ROS in pollen on olive stigma at stage 4 with DCFH_2_-DA at high magnification. Click here for file

Additional file 5 3-D reconstruction of CLSM detection of ROS in olive anther at stage 4 with DCFH_2_-DA at low magnification. Click here for file

Additional file 6 3-D reconstruction of CLSM detection of superoxide anion in olive flower at stage 3 with DHE at low magnification. Click here for file

Additional file 7 3-D reconstruction of CLSM detection of superoxide anion in olive stigma at stage 4 with DHE at low magnification. Click here for file

Additional file 8 3-D reconstruction of CLSM detection of superoxide in olive anther at stage 4 with DHE at low magnification. Click here for file

Additional file 9 z-Animated 3-D reconstruction of CLSM detection of superoxide in pollen on olive stigma at stage 4 with DHE at high magnification. Click here for file

Additional file 10 3-D reconstruction of CLSM detection of NO in pollen on stigma surface at stage 3 with DAF-2 DA at medium magnification. Click here for file

Additional file 11 3-D reconstruction of CLSM detection of NO in pollen on stigma surface at stage 3 with DAF- 2 DA at high magnification. Click here for file

Additional file 12 z-Animated 3-D reconstruction of CLSM detection of NO in pollen on stigma surface at stage 4 with DAF-2 DA at high magnification. Click here for file

Additional file 13 3-D reconstruction of CLSM detection of NO in pollen on stigma surface at stage 4 with DAF-2 DA at high magnification. Click here for file

Additional file 14 3-D reconstruction of CLSM detection of NO in olive anther at stage 4 with DAF-2 DA at low magnification. Click here for file
